# Land Surface Temperature Response to Irrigated Paddy Field Expansion: a Case Study of Semi-arid Western Jilin Province, China

**DOI:** 10.1038/s41598-019-41745-6

**Published:** 2019-03-27

**Authors:** Tingxiang Liu, Lingxue Yu, Shuwen Zhang

**Affiliations:** 1 College of Urban and Environmental Science, Changchun Normal University, Changchun, 130032 China; 2 Northeast Institute of Geography and Agroecology, Chinese Academy of Sciences, Changchun, 130102 China

## Abstract

The irrigated paddy fields have expanded greatly at semi-arid western Jilin province of China in recent over ten years, the sources of which are rain-fed cornfields, swamp meadow and saline alkali land mainly. Based on regional land use data, remote sensing data and meteorological data, this paper evaluates the land surface temperature changes response to land surface biophysical processes changes resulting from land use change (LUC), and dissociates the effect of radiative change (albedo) and non-radiative change (evapotranspiration and turbulent process) quantitatively using the energy redistribution factor. The results show that, the total land surface temperature changes based on energy redistribution factor are consistent with that based on remote sensing data on the whole, which have significant and different seasonal variations for agriculture adjustment of rain-fed cornfields to irrigated paddy fields and nature land reclamation. Generally, the largest Land surface temperature changes (ΔTs) are most pronounced in May and June for agriculture adjustment of rain-fed cornfields to irrigated paddy fields, which is −1.85 K averagely. Notable decline of albedo from saline alkali land to irrigated paddy fields in April to June greatly counteracts the cooling effect of non-radiative processes changes, while the largest ΔTs is found of −2. 54 K in dry summer months of July and August. For swamp meadows to irrigated paddy fields, non-radiative process is strengthened from June to September, the cooling effect of which is −1.69 K averagely. This study provides a case reference of local temperature change and obvious changes of land surface non-radiative terms at semi-arid area for adjustment of agricultural activities and land use changes.

## Introduction

Land use changes (LUC), which have been traced to the pre-industrial age and have recognizable effects on climate systems at different scales ranging from the local to regional and global, have received much attention in recent decades^1–4^. In some regions, the impacts of LUC on temperature showed warming effects just like the effects caused by CO_2_ increasing, while in others, the opposite effect was observed^5–9^. In this way, LUC can dampen or enhance the impacts of increasing CO_2_. The impacts of LUC can be more significant at the local and regional scales, which could create important disturbances at larger scales and even affect the global climate system^4,10,11^.

Transformations of the Earth’s surface fundamentally alter the fluxes of solar and thermal infrared radiation, sensible and latent heat, the movement of water between the sub-surface and atmosphere, and the exchange of momentum between the land surface and atmosphere^4,12,13^. LUC-induced changes of surface biogeophysical characteristics, such as albedo^14^, surface roughness^15^, and Bowen ratios^16,17^, have direct impacts on the heterogeneity and quantity of local temperature, humidity and wind speed, which make biogeophysical processes critical to the local climate system^4,18^. At present, worldwide land cover changes have received extensive attention due to their significant effects on large-scale climate, including changes in deforestation and afforestation^19–22^, desertification^23^, and snow and ice cover^24^. Meanwhile, meso-micro-scale climate changes resulting from typical LUC are similarly notable and may even be stronger than background climate change^10,25–27^. These local and regional LUC include urbanization and wetland shrinkage, as well as paddy land expansion, though research on the latter has not attracted significant attention.

Paddy land expansion is one of the land use adjustments caused by agricultural planting structures or reclamation work, and it generally occurs in areas where water resources are relatively abundant. However, the construction of artificial irrigation facilities makes it possible to plant large areas of rice in semi-arid regions if the nearby water resources are sufficient. Paddy land expansion in semi-arid areas leads to the land surface transforming from one with a water shortage directly to a humid state during the crop growth period, and further results in a significant increase in regional atmospheric humidity and a reduction of near-surface temperatures due to the significant increase of latent heat flux primarily from the energy exchange process^28^. This phenomenon was defined as the “cold-humid effect”, which is essentially the opposite of the “warming and drought effect” resulting from wetland shrinkage^29,30^. Both of these effects form heterogeneous land surface processes and greatly enhance the complexity of the local and regional climate system.

Previous assessment and simulation studies on the relationship between land cover changes and surface-level biophysical processes have not been widely recognized, mainly due to the lack of comprehensive observation support^31,32^. For a given ecosystem or land cover type, *in situ* measurements of energy fluxes and other meteorological variables are necessary to understand how radiative energy is channeled into latent heat and how sensible heat is dissipated away from the surface^11,33^. Some researchers have formalized approaches to estimate a discrete change in the surface temperature by rearranging the terms of the surface energy balance and taking first-order derivatives to assess radiative and non-radiative terms in isolation^15,34,35^. For instance, Lee (2011) formulated an expression combining Fick’s law of diffusion with Bowen ratios as a combined measure of the aerodynamic and physiological controls governing surface energy budgets, which was termed the energy redistribution factor^15^. Based on this research, Bright *et al*.^17^ combined predictions from a semi-mechanistic empirical model with satellite remote sensing and other global observations to provide global estimates of the local direct surface temperature response to several land cover and land management changes, which provided a means to efficiently map and attribute local surface energy balance responses to multiple land cover types, as well as a feasible method for evaluating the effect of land cover change in climate models^11^.

In this paper, taking the semi-arid western region of Jilin Province in China as a study area, we combine the energy redistribution factor model with moderate-resolution imaging spectroradiometer (MODIS) data and other observation data to estimate the local land surface temperature change response to paddy land expansion and to analyze the spatial and temporal variations in this process. This study reveals the contribution rates of radiative processes and non-radiative processes, mainly albedo and evapotranspiration, respectively, on land surface temperature change in semi-arid areas, and further provides a basis for agricultural climatic resource changes and corresponding adjustments of agricultural activities.

## Results

### Irrigated paddy field expansion

**Figure 1:**
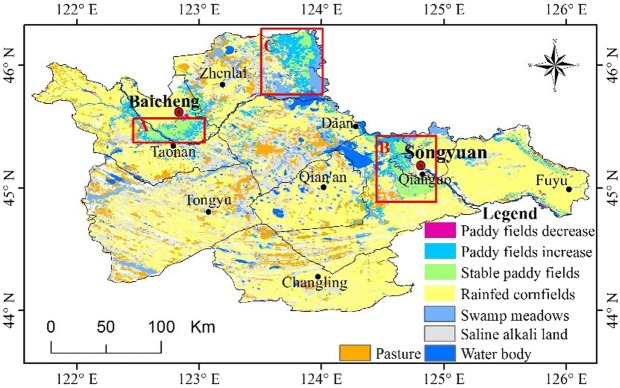
Irrigated paddy field dynamic of western Jilin Province, China, 2005–2017.

Based on the land use data from 2005 and 2017, the expansion of irrigated paddy fields is represented in Fig. 1.

In recent years, the irrigated paddy fields of western Jilin Province have increased from 166 thousand hectares in 2005 to 400 thousand hectares in 2017, with an average annual growth rate of 7.6%. The paddy fields are mainly located in three regions, which are zone A, zone B, and zone C, as shown in Fig. 1. For these new paddy fields, 44.5% come from rain-fed cornfields due to the adjustment of agricultural activities, and 39.9% are attributed to the reclamation of unused land (including 21.7% of swamp meadows and 18.2% of saline alkali land). According to the land use data, there is also a small amount of paddy fields that are abandoned, which are located at the edge of the saline alkali land.

### Land surface energy redistribution change for paddy field expansion

**Figure 2:**
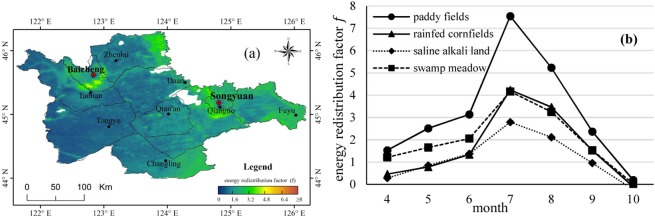
Mean energy redistribution factor *f* from April to October. (**a**) Spatial distribution of monthly mean *f*, excluding water surface (blank area within study region). (**b**) Monthly variations of *f* for different land types.

We focus on the land surface energy redistribution associated with agricultural activity; the average energy redistribution factor *f* is computed from April to October, as shown in Fig. 2(a), with monthly variations of *f* for rain-fed cornfields, swamp meadows, saline alkali land and irrigated paddy fields shown in Fig. 2(b). The reason for choosing the period of April to October is that the crops in rain-fed cornfields and irrigated paddy fields usually be planted at the end of April and harvested at early October. Agriculture land surface would be very different from the others from April to October without snow cover. In this paper, considering the valid contrast between land use types as well as a sufficient number of sample grids, pixels that had land use percentages higher than 95% in each 1-km pixel and unchanged during 2005 to 2017 were selected as samples in the subsequent analysis, which are close to the paddy fields of zone A, zone B, and zone C in Fig. 1. The sample number of paddy fields, rain-fed farmland, swamp meadows and saline alkali land are 591, 2916, 489 and 524 respectively.

When comparing Figs 2 with 1, we find that *f* is typically higher for irrigated paddy fields compared to other lands. The *f* value differences were statistically significant at a p < 0.05 significance level based on Welch’s t-test. The paddy field expansion leads to a significant rise in *f*. This higher value corresponds to higher efficiency in non-radiative processes, either via evapotranspiration or the turbulent exchange of sensible heat, and is generally associated with lower temperature gradients at the surface-atmosphere boundary.

For the monthly variation, the peak value of *f* is in July for every land use type, while the second-highest value is in August, which indicates the great efficiency of evapotranspiration or turbulent exchange in July and August. All *f* values increase from April to July, including a sharp rise from June to July, indicating enhancement of evapotranspiration or turbulent exchange between land surface and the air, especially from June to July. The reasons would be the increase of ground heat, soil water content, and vegetation transpiration makes land non-radiative processes become stronger. The *f* value decreased from August to October, because the decrease of temperature, precipitation and vegetation transpiration makes land non-radiative processes continuously reduce from high level to very small. The seasonal differences between the four land use types rely on the soil moisture and seasonal vegetation cover differences. The higher *f* values for paddy fields indicate that irrigation plays an important role in artificially elevating the evapotranspiration, both from the evaporation of bare land surfaces before crop closure and for crop transpiration after crop closure. In July and August, *f* is slightly higher for rain-fed cornfields than for swamp meadows, which shows that the evapotranspiration efficiency for cornfields may be slightly greater than that for local swamp meadows in the semi-arid area. From April to June, *f* is higher for swamp meadows than that for rain-fed cornfields and saline alkali land, which may be the result of a similar shortage of vegetation coverage and soil water content for the latter two categories in this period.

### Land surface temperature change response to land use transition

**Figure 3:**
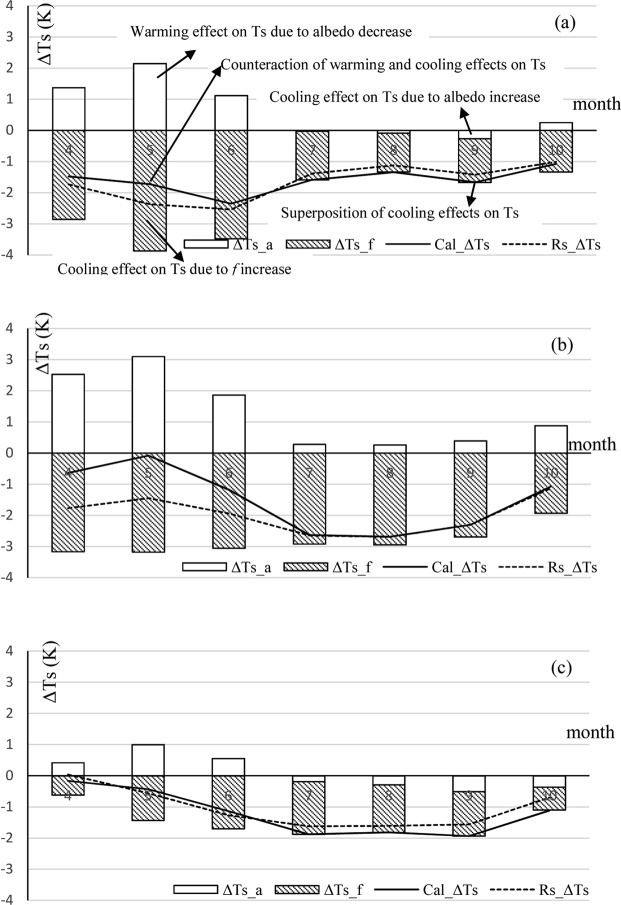
ΔTs based on remote sensing data (Rs_ΔTs) and the energy balance equation (Cal_ΔTs), and contribution of albedo change (ΔTs_a) and energy redistribution factor change (ΔTs_f), between rain-fed cornfields (**a**), saline alkali land (**b**), swamp meadows (**c**) and irrigated paddy fields, respectively.

Land surface temperature changes (ΔTs) in land use transitioning from rain-fed cornfields, swamp meadows and saline alkali land to irrigated paddy fields are calculated based on Equation (4), as well as ΔTs partly resulting from albedo and non-radiative forcing process changes, respectively. The total ΔTs values calculated based on Equation (4) are also compared with the ΔTs values derived from remote sensing data (Fig. 3).

As represented in Fig. 3, the variations of Rs_ΔTs and Cal_ΔTs are basically consistent with each other, which indicates that ΔTs_a and ΔTs_f are acceptable. According to the land surface energy balance process, in land transitioning to irrigated paddy fields, the resulting decrease of albedo induces warming effects while the increases in evapotranspiration and turbulent processes correspond to cooling effects. Figure 3(a) shows that when coupling the warming effect with the cooling effect, the transition from rain-fed cornfields to irrigated paddy fields produces an average net decrease of −1.85 K (Cal_ΔTs) from April to June. From July to September, the slightly changed albedo no longer counteracts the cooling effect, while the difference of evapotranspiration and turbulent process between two farmlands is weaker than before, which produces an average net decrease of −1.5 K (Cal_ΔTs). Figure 3(b) demonstrates that for the transition from saline alkali land to irrigated paddy fields, the quantity of the warming effect is slightly less than that of the cooling effect from April to June, which results in a less significant net decrease of no more than −1 K (Cal_ΔTs) on average. As the decrease of the warming effect and the stabilization of the cooling effect occurs from July to September, the net decrease of ΔTs reaches −2.54 K (Cal_ΔTs) on average. Comparing Fig. 3(c) with the former two figures reveals that the cooling effect in June to September is typical, while the degree of this cooling is between those of the former two transitions in the same period, that is, −1.69 K (Cal_ΔTs) on average. The temperature change values above were statistically significant at a p < 0.05 significance level based on Welch’s t-test.

## Discussion

### The drivers of irrigated paddy field expansion

In the pursuit of economic interests and ecological benefits, numerous rain-fed cornfields, swamp meadows, and saline alkali land have been converted or reclaimed for use as irrigated paddy fields. Benefitting from the newly built diversion irrigation facilities adjacent to the rivers in the recent years, the rain-fed cornfields and swamp meadows in this semi-arid area can be successfully converted to paddy fields. Additionally, the improvement measures and new technologies in agriculture have been used to cultivate rice on saline alkali land.

The areas where the irrigated paddy fields are concentrated is a lowland alleviated by several rivers, which were previously areas for rice production. In the past years, the “temporary purchase and store policy” for corn in China has increased the enthusiasm of farmers to convert the paddy fields to plant corn in order to obtain more economic value. As a result of the excessive corn production capacity, the corn purchase policy has changed, and expected earnings from planting corn are now significantly lower than those from planting rice. As a result, numerous farmers have converted their rain-fed cornfields to irrigated paddy fields, which has contributed to 44.5% of the total paddy field increase. Furthermore, the land use change from swamp meadows to paddy fields was driven by the profit improvement of planting rice. The uncultivated swamp meadows in lowlands adjacent to the rivers, such as region C in Fig. 1, have become the new sources for paddy field expansion and accounted for 21.7% of the newly increased paddy field area.

To utilize the wide-spreading saline alkali land in the study area, some researchers have developed a series of measures, including soil improvement, variety breeding, cultivation, and irrigation technologies, to plant rice on saline alkali land. These measures have improved the physiochemical properties of the saline alkali land surface, significantly improving the ecology and functionality of the local landscape. As a result, the production function has recovered in these areas as well. However, this land use approach has not been promoted extensively due to its cost. According to land use data, the paddy fields converted from saline alkali land are dispersed at the edge of the saline alkali land, which is unstable and may be abandoned in the coming years. Moreover, the climate background in this region has become warmer and drier^36,37^, which contradicts the continuous water resource requirements of paddy fields. Therefore, it is essential to conduct risk assessments of paddy field expansion.

### The estimation of the energy redistribution factor

In this study, the estimation of the energy redistribution factor for each land use type is the key issue. In a study conducted by Bright *et al*.^11^, researchers first calculated the regional *f* values; then, global upscaling was conducted using the radiation, temperature and precipitation variables derived from remote sensing, meteorological datasets and reanalysis data. Our *f* values for rain-fed farmland show no significant differences compared to those obtained by Bright. From Equation (1), we can find that (Ts-Ta) dominated the magnitude of *f* values and showed significant negative correlations, while Rn* showed no obvious variations. This means that the larger the difference between land surface temperature and the air temperature is, the more energy will be emitted from the surface through surface longwave radiation other than through latent and sensible heat exchanges between the surface and atmosphere, resulting in a weak non-radiative process and small *f* values, and vice versa. In Lee’s study, the Bowen ratio was calculated based on observations, and they used the S9 in reference from^15^ to estimate the f value. However, (Ts-Ta) is still the dominant factor in the calculation of the Bowen ratio, indicating that even though the energy redistribution factor can effectively represent radiative and non-radiative processes, the precondition is that the near-surface air heat comes from the land surface completely, which is characterized by Ts > Ta. In other words, when the Ts is much closer to Ta or the Ts is less than Ta, the *f* values lack physical explanations.

The Equation (4) used in this study was modified based on the S10 from a reference of Lee *et al*.^15^. On the local scale, since we have already obtained the f values for the two land cover types, the denominator of the second section of the right-hand terms can be estimated by $(1+f)(1+f+{\rm{\Delta }}f)$ instead of ${(1+f)}^{2}$. When f changes significantly due to land cover change, such as the conversion of rain-fed cornfields to irrigated paddy fields, this modification can help us obtain a ΔTs value that is closer to the observations. In our analysis, the MODIS land surface temperature data are the key data resource. Although there are limitations for the land surface temperature change research based on remote sensing^11,21^, our results (Fig. 3) show that the land surface changes extracted by remote sensing are consistent with the land surface temperature changes derived from the energy balance method overall.

### The temperature responses to irrigated paddy field expansion

Each block of land has an independent energy budget process, which varies with vegetation (crops)^29^. The land surface temperature is the purest measure of the composite biogeophysical attributes of the land surface and their controls on its energy balance^38^. A number of observational and simulation studies have documented the cooling effect caused by the irrigation on farmland^39–41^. In this study, we focused on the surface temperature responses to the irrigated paddy fields expansion and showed seasonal variation such as those found in Kueppers’s study^40^. Kueppers illustrated that the largest temperature differences between irrigated farmland and natural vegetation appeared in the summer months, i.e., July and August. However, in our results, the maximum surface temperature differences between irrigated paddy fields and rain-fed farmland occurred in May and June, while the largest differences between the paddy field and the saline alkali land as well as swamp meadows mainly appeared in July, August and September. This suggests that the effective mechanisms between the cultivation from natural vegetation to irrigated farmland and the conversion from rain-fed farmland to irrigated paddy fields are different and should thus be separated in the model simulations or the observations.

The irrigated paddy fields in the study area require standing water in May and June, which makes the surface temperature decrease significantly due to strong evapotranspiration. On the other hand, the rain-fed cornfields have much lower transpiration because of their low vegetation coverage in this season under semi-arid climate conditions. In July or August, the standing water disappears in the paddy fields, while the vegetation coverage can reach almost 100% in both the paddy fields and rain-fed cornfields, resulting in less difference in land surface processes between the two crops compared to May or June. This characteristic and its corresponding biophysical process showed significant differences compared with the paddy fields of other regions in China. For example, in studies based on water balance and transport, Puma and Cook^42^ proposed that the evapotranspiration of most paddy fields in China showed no significant increase compared with the surrounding natural vegetation, suggesting that the constraining factor of evapotranspiration was energy instead of moisture. The climate condition in our study area is relative arid, making the soil moisture become the main constraint affecting evapotranspiration in land without irrigation.

In addition to evapotranspiration, the changes in albedo introduce a warming effect to the local temperature in the process of paddy field expansion, indicating that the lower albedo can help the surface absorb more shortwave radiation and warm the surface in typical rain-fed cornfields and saline alkali land. From April to June, when there is little soil moisture and vegetation coverage in rain-fed cornfields and saline alkali land, as well as standing water or significant soil moisture in paddy fields, the significant difference in albedo can dampen the cooling effects caused by the non-radiative process to a certain extent. In July or August, the evapotranspiration in paddy fields is obviously higher than that in saline alkali land while the albedo difference is relatively small, resulting the most significant temperature decrease.

Overall, the mean land surface temperature differences between the three land types converted to irrigated paddy fields do not have a great disparity. According to land surface temperatures derived from MODIS data, we find that for irrigated paddy fields, the daytime average Ts is lower, while the nighttime Ts is slightly higher than in rain-fed cornfields in May and June. With radiative forcing occurring in the daytime and vanishing at night, while non-radiative processes persist all the time, the daytime and nighttime variations in non-radiative processes should be analyzed in future studies, especially for rain-fed cornfields converted to irrigated paddy fields.

## Conclusions

Although they experience little precipitation and large amounts of evaporation, irrigated paddy fields have expanded greatly in western Jilin Province over the last ten years, and they were mainly converted from former rain-fed cornfields, swamp meadows and saline alkali land. The standing water and elevated soil moisture in irrigated paddy fields greatly increase the latent heat flux between the land surface and atmosphere, which results in a significant decrease of land surface temperature compared to other land use types. The decrease of albedo induces a warming effect that counteracts the cooling effect to a certain degree, which mainly occurs from April to June before crop closure. Based on the energy redistribution factor, this paper evaluates the response of land surface temperature changes to these processes and quantitatively dissociates the effect of radiative change (albedo) and non-radiative change (evapotranspiration and turbulent process). The results indicate that in both rain-fed cornfields and natural lands, a shift in land use to irrigated paddy fields results in significant seasonal variations in land surface temperature change. Generally, the largest ΔTs are most pronounced in May and June for agricultural adjustment from rain-fed cornfields to irrigated paddy fields, which is −1.85 K on average. A notable decline in albedo from saline alkali land converted to irrigated paddy fields from April to June greatly counteracts the cooling effect of non-radiative process-based changes, while the largest ΔTs is found at −2.54 K in the dry summer months of July and August. For swamp meadows that have been converted to irrigated paddy fields, non-radiative processes are strengthened from June to September, the cooling effect of which is somewhere in between of the other two land use changes and averages −1.69 K. The total land surface temperature changes based on the energy redistribution factor (Cal_ΔTs) are consistent with those based on remote sensing data overall.

Paddy fields are mainly distributed in humid and semi-humid agricultural areas at middle and low latitudes all over the world, and this paper focuses on the irrigated paddy field expansion occurring in the inland semi-arid regions of the middle latitudes. Irrigated paddy field expansion from both natural lands and rain-fed farmlands not only affects the global climate through the carbon cycle but also changes the local temperature more significantly, which is typical in the case of intensive climate change responses to human activities. This study provides a local reference for the comprehensive evaluation of the multi-scale impacts of agricultural land use management.

## Materials and Methods

### Study area

The study area (43°59′27″–46°18′5″N, 121°37′31″–126°10′43″E) is an inland region far from the coast, primarily including the cities of Baicheng and Songyuan in Jilin Province, China. This region experiences a semi-arid continental monsoon climate in the middle latitude temperate zone. The annual precipitation is 300–400 mm, which is much less than the potential evapotranspiration. This area belongs to the agro-pastoral transitional zone, which is a fragile ecological environment. The western region of Jilin Province has large areas of unused saline alkali land and abundant water resources, with several rivers passing through it, including the Taoerhe, Nenjiang, and Second Songhua rivers. Under the leadership of the local government, a “General Building Plan of Improving Commodity Grain Production Capacity” has been implemented to increase grain production from 2.5 × 10^10^ kg to 3.0 × 10^10^ kg in five years or longer, which motivated the development of irrigated paddy fields from rain-fed cornfields, swamp meadows, and saline alkali land. In general, the crop-growing season spans from May to October in the study area, while both rain-fed and irrigated farmland would be plowed and prepared for seeding in April.

### Methods

Land surface temperature is greatly affected by the pattern of how the radiative energy is channeled into latent heat, and how sensible heat is dissipated away from the surface^11^. The energy redistribution factor (*f*) was proposed as a combined measure of the aerodynamic and physiological controls governing surface energy budgets^15^. The higher value of energy redistribution factor corresponds to higher efficiency in non-radiative processes and is generally associated with lower temperature gradients at the surface-atmosphere boundary. Based on the linearization of the surface longwave radiation term in the surface energy balance equation, the energy redistribution factor *f* is introduced as^11,15,43^:

1 $$f=\frac{{\lambda }_{0}}{\,{T}_{s}-{T}_{a}}({R}_{n}^{\ast }-G)-1$$

where *T*_*s*_ is the monthly mean surface radiometric temperature (K); *T*_*a*_ is the monthly mean air temperature (K) at 2 m above the reference height; *λ*_0_ = *1/(4σε*_*s*_*T*_*s*_^3^), the temperature sensitivity resulting from the longwave radiation feedback, the range of which showed slightly variations with the surface temperature changes, and *ε*_*s*_ is the monthly mean surface emissivity; ${R}_{n}^{\ast }=S+{L}_{\downarrow }-\sigma {T}_{a}^{4}$ is the monthly mean apparent net radiation; *S* is the net shortwave radiation; ${L}_{\downarrow }$ is the incoming longwave radiation; and G is the monthly mean soil heat flux.

In regional or local studies, the difference of net shortwave radiation on two land cover sites is mainly based on surface albedo, while incoming longwave radiation and soil heat flux are slightly affected by land cover type. Thus, in this paper, net shortwave radiation (S) and soil heat flux (G) are estimated by an empirical formula^44^:

2 $${\rm{S}}=(1-{\rm{\alpha }})(0.25+0.5{\rm{r}})37.586d(\psi \,\sin \,\phi \,\sin \,\delta +\,\cos \,\phi \,\cos \,\delta \,\sin \,\psi )$$

3 $${\rm{G}}=0.14({T}_{a,n}-{T}_{a,n-1})$$

where α is the surface albedo, r is the percentage ratio of sunshine duration in each month, d is the relative distance from the Earth to the Sun, $\psi$ is the sunset hour angle (rad), $\phi$ is the latitude (rad), and $\delta$ is the solar declination (rad). Finally, the units of S and G are converted to W m^−2^.

Considering that local paddy land and other surrounding lands share the same background climate, and ignoring changes in G, the surface temperature response to land use change is approximated^11,15^:

4 $${\rm{\Delta }}{T}_{s}=\frac{{\lambda }_{0}}{(1+f)}{\rm{\Delta }}S+\frac{-{\lambda }_{0}}{(1+f)(1+f+{\rm{\Delta }}f)}({R}_{n}^{\ast }-G){\rm{\Delta }}f$$

Where *λ*_0_, *f*, ${R}_{n}^{\ast }$, G are variables of land use type before land use change, while ${\rm{\Delta }}S={\rm{\Delta }}{R}_{n}^{\ast }$, and $\Delta f$ are variable changes resulting from land use change.

Based on Eq. (4), the land surface temperature change is calculated, and the first and second terms on the right side of Eq. (4) represent radiative forcing associated with albedo changes, energy redistribution associated with roughness changes and Bowen ratio changes, respectively.

## Data Availability

The expansion of paddy land is described through the change in land use data over two periods, including 2005 and 2017. The land use data from 2005 were obtained from the Chinese land use/land cover database “Integrated Scientific Expedition in North China and Its Neighboring Area Project”^45^ based on Landsat 5 Thematic Mapper (TM) images, while the data from 2017 were regenerated with interactive interpretation based on the land use data from 2005 and Landsat 8 Operational Land Imager (OLI) images (https://glovis.usgs.gov/) from 2017. The detailed regenerating procedure can be found in Liu *et al*.^46^. We used the Daily Moderate Resolution Imaging Spectroradiometer (MODIS) albedo product (MCD43A3.V006) to calculate the land surface albedo (*α*) in our study area. We also used the 8-day composite MODIS land surface temperature product (MYD11A2.V006) to obtain the land surface temperature for our study area (*Ts*) and surface emissivity (*ε*_*s*_) (https://search.earthdata.nasa.gov/). All of these data are computed as monthly mean values from 2005–2017. The monthly climatologies of *T*_*a*_ and r are spatially interpolated using the meteorological observation data of the China Meteorological Data Service Center (http://data.cma.cn/en). The monthly ${L}_{\downarrow }$ is extracted from the reanalysis data of MERRA-2 (Modern Era Retrospective Analysis for Research and Applications, Version 2) (https://disc.gsfc.nasa.gov/daac-bin/FTPSubset2.pl).
